# Can a Scaffold Enriched with Mesenchymal Stem Cells Be a Good Treatment for Spinal Cord Injury?

**DOI:** 10.3390/ijms23147545

**Published:** 2022-07-07

**Authors:** Santino Blando, Ivan Anchesi, Emanuela Mazzon, Agnese Gugliandolo

**Affiliations:** IRCCS Centro Neurolesi “Bonino-Pulejo”, Via Provinciale Palermo, Contrada Casazza, 98124 Messina, Italy; santino.blando@irccsme.it (S.B.); ivan.anchesi@irccsme.it (I.A.); agnese.gugliandolo@irccsme.it (A.G.)

**Keywords:** spinal cord injury, mesenchymal stem cell, scaffold, fibrin, collagen, chitosan, PLGA

## Abstract

Spinal cord injury (SCI) is a worldwide highly crippling disease that can lead to the loss of motor and sensory neurons. Among the most promising therapies, there are new techniques of tissue engineering based on stem cells that promote neuronal regeneration. Among the different types of stem cells, mesenchymal stem cells (MSCs) seem the most promising. Indeed, MSCs are able to release trophic factors and to differentiate into the cell types that can be found in the spinal cord. Currently, the most common procedure to insert cells in the lesion site is infusion. However, this causes a low rate of survival and engraftment in the lesion site. For these reasons, tissue engineering is focusing on bioresorbable scaffolds to help the cells to stay in situ. Scaffolds do not only have a passive role but become fundamental for the trophic support of cells and the promotion of neuroregeneration. More and more types of materials are being studied as scaffolds to decrease inflammation and increase the engraftment as well as the survival of the cells. Our review aims to highlight how the use of scaffolds made from biomaterials enriched with MSCs gives positive results in in vivo SCI models as well as the first evidence obtained in clinical trials.

## 1. Spinal Cord Injury

Spinal cord injury (SCI) refers to a damage caused by compression, laceration, or contusion of the spinal cord that impairs its function. It has an incidence of 250,000–500,000 individuals every year [1]. The causes are divided into two types: traumatic SCI and non-traumatic SCI [2]. Traumatic SCI (TSCI) occurs when an external factor damages the spinal cord through a strong impact. Non-traumatic SCI (NTSCI) is the result of complex multifactorial pathological processes and is defined as any damage to the spinal cord not caused by a factor of a traumatic nature [3,4]. The primary injury of SCI is the initial mechanical damage, which can compromise the physiology of the spinal cord due to interruptions in the neuronal transmission [5]. A secondary injury is induced by the vascular and biochemical effects that result in an imbalance of the physiology of the multi-layered microenvironment. The primary injury triggers the secondary injury, which causes other damage to the spinal cord tissues, leading to excitotoxicity, an increase in glutamate, oxidative stress, increased cell permeability, apoptosis, ischemia, edema, neuroinflammation, demyelination, and fibroglial scar and cyst formation [1]. All these processes lead to neurological dysfunction. The secondary lesion begins and is divided into three phases: early acute, subacute, and chronic [1]. 

During SCI there is a high generation of free radicals, which cause oxidative stress, and lipid peroxidation, which contributes to the secondary injury process [6]. These events lead to neuroinflammation with blood–spinal cord barrier (BSCB) disruption, glial activation, and peripheral macrophage infiltration. Astrocytes at the lesion site produce cytokines and other pro-inflammatory factors and, together with activated microglia, produce several pro-inflammatory cytokines (IL-1a, IL-1b, and TNF-α), inducing the homing of peripheral macrophages to the lesion site [7]. 

The loss of neural tissue and extracellular matrix (ECM) causes the formation of cystic cavities where inflammatory cells can infiltrate, leading to the formation of the glial scar, a barrier that abolishes endogenous axonal regeneration. The glial scar limits axonal regeneration in conjunction with other inhibitory factors such as chondroitin sulfate proteoglycans (CSPG) [8]. An insufficient trophic or mechanical support can also inhibit regeneration. Then, it is necessary to create a permissive environment, reducing inflammation and axonal regeneration inhibitors and increasing neurotrophic factors to promote axonal growth [9]. Primary and secondary injuries carry a complex cascade of damaging events, and there are currently no strategies for the complete resolution of SCI. The acute phase is considered the optimal therapeutic window. 

Corticosteroids are drugs in that can reduce various cellular stresses caused by SCI, and some trials evidenced that they can also promote motor recovery. However, high doses can induce adverse reactions such as gastrointestinal bleeding, sepsis, and pulmonary embolism [10]. Some guidelines [11] highlight that, currently, the use of cortisone in the acute phases of SCI is widely used, although it is not indicated as a pharmacological treatment. 

Other therapeutic pathways include *N*-methyl-D-aspartate receptor (NMDA) and α-amino-3-hydroxy-5-methyl-4-isoxazolepropionic acid receptor (AMPA) inhibitors, such as gacyclidine and magnesium, which had neuroprotective effects in animal models [10]. 

In addition, neurosurgical procedures for the decompression and safe stabilization of the spinal cord are important to avoid the extension of damage in other areas, but this does not prevent the progression of the secondary lesion [12]. 

Given that there are currently no strategies for the complete resolution of SCI that are also able to induce nervous regeneration, researchers are looking for new therapeutic strategies. Studies have also shown that an intravenous infusion of mesenchymal stem cells (MSCs) after acute SCI may induce transient gene expression changes in the brain, with early functional improvements in SCI [13]. The mechanisms by which infused MSCs might contribute to gradual functional recovery are remyelination, immunomodulation [14], the replacement of injured cells [15], and the enhancement of neural plasticity [16]. However, the possibility of the formation of ectopic colonies at sites distant from the lesion site [17] led to the use of biomaterials capable of limiting cell migration. A promising therapeutic strategy for SCI is the use of stem cell therapy and biomaterials as scaffolds aimed to induce neuroprotection and regeneration, as the presence of the scaffold promotes cell survival and cellular differentiation [18]. In particular, among the different stem cell types, MSCs seem to induce therapeutic effects thanks to their ability to release their own secretome containing antiapoptotic, neurotrophic, and anti-inflammatory molecules [19]. 

In this review, we discuss studies that evaluated the therapeutic potential of scaffolds enriched with MSCs in in vivo SCI models. The focus was to highlight the cellular and functional effects in relation to the MSCs/scaffold co-graft, especially nerve regeneration and motor recovery. Only in vivo model studies were considered because only in vivo models can best simulate SCI. With this aim, we performed a Pubmed search, looking for studies published in the last 5 years in which MSCs were used in combination with different biomaterials. The keywords of the research were: “Spinal Cord Injury”, “Mesenchymal Stem Cell”, and the various types of scaffolds based on biomaterials. From our research, only four biomaterials were found: collagen, fibrin, chitosan, and poly(lactic-co-glycolic) acid (PLGA). The other biomaterials did not use MSCs, or the studies were not in vivo. With the possibility to translate the in vivo studies in humans in mind, we also discuss the first evidence obtained in clinical trials.

## 2. Biomaterials and Scaffold

Tissue engineering techniques combine scaffolds and cells. Over the years, this part of regenerative medicine is expanding, with more studies focusing on cell cultures in 3D to create a better model, encouraging the understanding of cellular mechanotransduction through cell–cell, cell–matrix, and cell–substrate interactions.

Scaffolds are three-dimensional structures that promote cell survival, cellular interactions, proliferation, physical protection, and ECM deposition. The scaffolds protect the cells when they are transplanted into a diseased or degenerated tissue. The scaffold must not cause damage such as inflammation or toxicity. In addition, it should also allow the transport of gas and nutrients to allow cell growth [20]. The scaffold can be made from bioabsorbable materials or from non-bioabsorbable materials. In the beginning, the scaffolds were non-bioabsorbable, and this caused the continuous onset of inflammation. Currently, a good scaffold should be totally bioabsorbable in order to not leave traces of its presence once its function is completed and to avoid a subsequent intervention. The disappearance of the totally bioabsorbable scaffold occurs through two processes: degradation in the case of an abiotic mechanism and biodegradation in the case of a cell-mediated mechanism. The biomaterials used to make the scaffolds should be biocompatible and must also have a certain mechanical resistance; they must be resistant to mechanical stress to support growing tissues. Ideally, biomaterials for SCI should also support axonal growth with an appropriate stiffness and provide a space through which axons can pass through or enter the scaffolds.

At present, injectable hydrogels have attracted the attention of researchers. Hydrogels are a 3D network of the same or different cross-linked hydrophilic polymers with a good water absorption capacity. Hydrogels can be combined with cells, growth factors, or drugs. The polymer matrix ensures proliferation, adhesion, and cell migration capacity, and the high water content aids nutrient diffusion [21]. Their most advantageous feature for SCI lies in the ease of implantation into the lesion, as they can be injected. After the injection, the quick transition that allows the liquid to become a gel also allows a better adaptation to the tissue at the injury site; thanks to this feature, the free spaces are eliminated, and a template for tissue regeneration is formed [22]. The gelation process occurs through a chemical or physical crosslinking. A hydrogel made with physical crosslinking denotes a gelation process without the participation of covalent bonds. Chemical crosslinking presents covalent bonds in their 3D network and increases the mechanical strength compared to a hydrogel made with physical crosslinking. The most used physical crosslinking methods are thermal condensation, molecular self-assembly, and ionic interactions, while the most used methods for chemical crosslinking are radical polymerization, Schiff base reaction, click reactions, and enzymatic crosslinking [23]. Some polymers can create different types of scaffolds based on the method used, for example, collagen can be in the form of hydrogel scaffolds or micro/nanofiber scaffolds [24]. The choice of how use the polymer greatly improves the coverage of use.

Nanofiber scaffolds are 3D porous devices with fibers with diameters in the nanometer range. Nanofibers have large surface-to-volume ratios, high porosity, and can be made relatively large. The 3D connectivity increases the effective surface areas and promotes chemical diffusion through the porous structures [25,26]. Scaffolds of this type have less water inside. Different methods can be used for the creation of nanofiber scaffolds, such as electrospinning, drawing, self-assembly, and thermal-induced phase separation. The most used method is electrospinning. This method uses an electric force to draw charged threads of polymer. Thanks to the simple electrospinning process and the affordable prices, electrospun scaffolds have received much attention. Electrospun fibrous scaffolds have remarkable advantages in both ECM-biomimetic structures and the ability to adsorb bioactive factors [25].

The biomaterials have different origins: they can be synthetic or natural. Generally, synthetic polymers are associated with better mechanical properties, such as stiffness, and the simplest fabrication techniques, such as electrospinning and 3D printing. Meanwhile, natural polymers, such as collagen and chitosan, have poor mechanical properties and have limited compatibility with fabrication techniques. However, they show good biocompatibility, biodegradability, and low toxicity [27]. 

Many of the biomaterials used to create a scaffold are also used for drug delivery, giving the possibility of adding an anti-inflammatory molecule to improve the surrounding microenvironment and promote proper functioning [28,29]. 

Biomaterials can also be combined, forming hybrid scaffolds that allow the advantages of the different single biomaterials to be combined and to overcome some of the disadvantages [30]. Hybrid scaffolds take the best features of the materials that make them up. For example, collagen combined with silk improves the mechanical characteristics of collagen [31,32]. Another combination uses two biomaterials: one with the purpose of protecting the cells, while the other has the purpose of drug delivery. Heparin sulphate is used in combination with collagen for it is capacity to increase the mechanical strength of the scaffold, besides playing an important role in neuroregeneration [33]. Hydrogels can also be created by combining chitosan and other elements, such as β-glycerophosphate [34], hydroxyethyl cellulose, collagen [35], and ammonium chloride [36], which results in better neuronal regeneration. In order to improve the drug delivery, chitosan or an acellular spinal cord scaffold can be associated with PLGA [37].

### 2.1. Collagen

Collagen is a family of fibrous glycoproteins found in the ECM. In vertebrates, collagen is the most popular protein, with 28 different types, and it can be found in almost all tissues. Type I is the most present, which creates skin, tendon, vasculature, organs, and bone. Type II participates in cartilage formation, type IV forms basal lamina, the epithelium-secreted layer of the basement membrane, and type V forms cell surfaces, hair, and placenta [38]. Collagen is the main fibrous structural protein in the body, making it an excellent candidate for tissue repair and regeneration. A problem that must be overcome is the lack of mechanical strength and structural stability upon hydration. For this reason, it is usually modified or combined with other biomaterials [39]. The peculiarity of collagen is the triple helix structure. All triple helical parts of the collagen molecules have the repeating glycine at every third position (Gly–X–Y)n, with X and Y each being one of the 21 amino acids [40]. Collagen is resistant to proteolysis, but the single-stranded regions are sensitive to matrix metalloproteinases (MMPs) such as MMP-1, MMP-2, MMP-8, MMP-13, and MMP-14. The degradation products of collagen types I–III induce the chemotaxis of human fibroblasts, which promotes the restoration of tissue structure and functionality [41]. Common sources include bovine skin and tendons, porcine skin, intestine, bladder mucosa, and rat tail. Collagen can be used in the medical field in various ways, for example, as a scaffold. Natural collagen-based biomaterial is commonly purified by decellularization and maintains the original tissue properties and ECM structure. Collagen can also be remodeled into aligned fiber (anisotropic) [42], which is important to influence the degree of cell–substrate contact, induce cell polarization [43], and guide cell motility [44,45]. In addition to its use as a hydrogel, collagen can also be used as a nanofiber scaffold that can be obtained using different methods of preparation. Nanofiber scaffolds give the possibility of directing axonal growth in SCI [46]. Due to their easily modifiable nature, collagen scaffolds could also be used for drug delivery.

### 2.2. Fibrin

Fibrin originates in the last phase of the coagulation cascade due to the effect of thrombin on fibrinogen, and its degradation is carried out by plasmin that circulates in the blood as the precursor plasminogen. Fibrin is a natural nanoscaffold that provides a temporary structure that facilitates cellular activities and the deposition of a new ECM. This feature makes it an excellent candidate for creating a scaffold. Fibrin scaffold is a hemostatic adhesive surgical material formed by the combination of a concentrated solution of fibrinogen and factor XIII with a solution of thrombin and calcium; the aim is to form a clot, simulating the final phase of the coagulation cascade. Thanks to the ratio between thrombin and fibrin, a scaffold can be created with the desired thickness of the fibers, number of branch points, porosity, and gel permeability [47].

At lower concentrations of thrombin, it will form a clot with thick fibers, few branch points, and large pores, while higher thrombin concentrations create clots with thin fibers, many branch points, and small pores. These parameters can be changed by varying the temperature, pH, salt concentration, and presence of other proteins [48]. A fibrinolysis inhibitor can be added to increase its duration. A fibrin scaffold can be used like a nanofiber but can also be injected as a liquid, and it solidifies in situ.

This scaffold is naturally predisposed to bind proteins and create a favorable environment for cells.

### 2.3. Chitosan

Chitosan is a cationic linear polysaccharide formed by randomly distributed β-(1→4)-linked D-glucosamine and N-acetyl-D-glucosamine. Chitosan can be obtained by the deacetylation of chitin with alkaline substances. The positive charge gives it several beneficial characteristics. The most important features are solubility, biodegradability, and biocompatibility as well as muco-adhesion and hemostasis [49]. Moreover, it can also show anti-inflammatory and antioxidant properties [49]. The biodegradability in living organisms depends primarily on the molecular weight of the polymer and its deacetylation degree (DD). A higher DD increases the number of positive charges, which increases the interaction between chitosan and cells [50]. Chitosan can be biodegraded into non-toxic residues by lysozyme, which hydrolyses glucosamine-glucosamine, glucosamine-n-acetyl-glucosamine, and n-acetyl-glucosamine-n-acetyl-glucosamine linkages. Today, chitosan is used extensively for drug delivery thanks to its positive charge that allows interaction with the cell wall, improving its penetration [28].

Chitosan is a good candidate for the creation of a scaffold because it promotes cell adhesion and because the positive charge interacts with the various negatively charged proteins and glycolipids on the surface of cells, favoring the creation of a useful environment for the cells [51]. Another great potential of chitosan lies in the fact that this material can be found in various forms or shapes, including filaments, films, gels, sponges, bioactive fibers, nanofibers, filaments, coating mesh, and porous structures. The ability to orient neuronal growth by giving a specific shape to the scaffold is an extremely important feature for SCI treatment. The scaffold can be prepared in two ways: one as a chitosan-based dried scaffold and one as a hydrogel. This choice greatly improves the coverage of use of chitosan [52]. 

### 2.4. Poly(lactic-co-glycolic) Acid (PLGA)

PLGA is a biodegradable aliphatic polyester copolymer of lactic acid (LA) and glycolic acid (GA), whose forms are usually identified by the ratio between these two monomers [53]. PLGA is used in the medical field with several applications, such as in second-generation drug delivery stents. This is due to its characteristics of biocompatibility and biodegradability. Biodegradation occurs mainly through hydrolysis but also occurs through enzymes. 

The degradation of these polymers occurs in two ways: the first is the cleavage of the polymer chain to form oligomers first and then monomers; the second concerns the physical phenomenon of mass loss due to monomers and oligomers that spread out from the polymer. These phenomena depend on the grade of hydrophilicity, the type of chemical bond, and the grade of crystallinity, which is crucial since the crystalline domains, being less permeable to the penetration of water, slow down the hydrolysis process. Hydrolysis has two phases: in the first one, the diffusion of water molecules occurs inside the polymer, while in the second the actual hydrolysis reaction takes place. The most important factor to be considered regarding PLGA degradation is its composition. It is well-known that different ratios of LA:GA influence the rate of PLGA degradation, and this is mainly due to the different hydrophilic profiles of each monomer. GA is more hydrophilic than LA, so PLGA with higher proportions of GA is more hydrophilic, and consequently the degradation in vivo is faster [54]. InVivo Therapeutics Corporation created the neuro-spinal scaffold made of poly (lactic-co-glycolic acid)-b-poly-(L-lysine) (PLGA-PLL), two biocompatible and bioresorbable polymers. The scaffold is implanted into the gap in the spinal column, aiming to create a neuropermissive matrix that allows cells to heal and respond to the injury site and begin filling the cavity.

## 3. Mesenchymal Stem Cells

In 1991, Dr. Arnold I. Caplan isolated a class of multipotent cells from human and mammalian bone marrow and periosteum with the ability, in vitro, to be induced and differentiated into mesodermal tissue cells. These cells have been defined, for these characteristics, as “mesenchymal stem cells” [55]. According to the International Society for Cellular Therapy, the criteria for defining MSCs are: the ability to adhere to plastic in culture; the expression of CD105, CD73, and CD90; and the lack of CD45, CD34, CD14, CD11b, CD79alpha, CD19, and HLA-DR surface molecules. Furthermore, a peculiar feature is the ability to differentiate in vitro into osteoblasts, adipocytes, and chondroblasts [56]. In 2019, they added other features to identify MSCs, such as tissue origin, quantitative RNA analysis of selected genes, cell surface marker analysis, and secretome analysis [57].

MSCs have been isolated from different tissues: bone marrow, adipose tissue, skin, foreskin, salivary glands, limb buds, dental tissues, menstrual blood, and perinatal tissues. For their isolation, an enzymatic isolation protocol, an explant, or a combination of both are applied [58]. In vitro, these cells have the ability to differentiate into osteocytes, chondrocytes, and adipocytes, but according to various studies, MSCs, cultured under specific conditions and with an appropriate differentiation medium, have the ability to differentiate into neuron-like cells [59]. Given that bone-marrow-derived stem/stromal cells (BM-MSCs) were the first to be discovered, it is not surprising that BM-MSCs were among the first cells used for the treatment of SCI [60], even if they are considered the best MSC source for osteogenesis and chondrogenesis [61]. The MSCs mainly differentiated into neurocytes are BM-MSCs and adipose-derived stem cells (AD-MSCs) [62]. AD-MSCs present advantages compared to BM-MSCs. Indeed, they can be collected in subcutaneous fat, which is easily accessible compared to bone marrow, and are also more concentrated than BM-MSCs [63].

The study by Sonja Prpar Mihevc et al. demonstrates that AD-MSCs are promising in the field of central nervous system regenerative medicine. Indeed, AD-MSCs cultured with various growth media (KEM, NIMa, NIMb, and NIMc) and neurogenic inducers, such as B27 supplement, valproic acid, forskolin, N2 supplement, and retinoic acid, expressed neural markers, including tubulin beta III (TUBB3), neurofilament heavy (NF-H), microtubule-associated protein-2 (MAP2), and typical neuronal morphology, confirming differentiation into neurons and glial cells [64]. Moreover, when induced, AD-MSCs produced more neuron-like cells compared to BM-MSCs [62]. A more recently discovered source of MSCs is represented by dental tissues. These cells have typical MSC characteristics and self-renewal properties and possess mesodermal trilineage multipotency (osteocytes, adipocytes, and chondrocytes) [65]. If induced, they can be differentiated into neural cells [66]. Given their origin from neural crest, they are more prone to differentiate into neural cells [67]. 

The therapeutic ability of MSCs may also depend on their secretome in addition to their differentiation capacity. The secretome of MSCs contains soluble proteins, free nucleic acids, lipids, and extracellular vesicles [68]. MSCs can secrete protective and promoting factors for neural regeneration such as brain-derived growth factor (BDNF), vascular endothelial growth factor (VEGF), glia-derived nerve growth factor (GDNF), insulin-like growth factor-1 (IGF-1), ciliary neurotrophic growth factor (CNTF), nerve growth factor (NGF), and neurotrophic 3 (NT-3) [69]. For these peculiarities, MSCs are at the center of much research on SCI therapy, giving impetus to cell therapy and regenerative medicine. Furthermore, MSCs modulate the immune system and regulate the immune responses of many diseases because they influence the proliferation of T cells and the activity of B cells and Tregs and inhibit the activation of natural killer (NK) cells and regulate the balance of Th1 and Th2 [70]. The immunoregulatory action of MSCs occurs by cell–cell contact and through the secretome. 

MSCs are currently considered promising for regenerative therapy in SCI for their cell signaling capabilities via the secretome and for their differentiation capabilities.

## 4. Combined Approaches of MSCs and Scaffolds

The combination of scaffolds and cells is giving better and better results for the regeneration of SCI. A lesioned spinal cord is naturally characterized by the loss of cells due to the injury and the subsequent necrosis of the surrounding tissues. Among the main concerns for the further application of stem cells are the low survival rate and the uncontrollable migration or differentiation when administered, which is also due to the unfavorable microenvironment at the site of damage. The MSCs are excellent candidates for SCI treatment because they can transdifferentiate into neuron-like and glial-like cells [71] and can release trophic factors that induce tissue regeneration.

Neuroinflammation plays a main role in SCI and MSCs thanks to their immunomodulatory role, which may exert beneficial effects. The combined MSCs and scaffold should also promote the migration and differentiation into neurons of the patients mesenchymal cells and inhibit the glial scar tissue that impedes axon regrowth at the SCI lesion site. A key consideration for the scaffold is the physical features of the ECM to promote a regenerative environment, differentiation, and trophic support. In comparison to other natural living tissues, the elastic modulus of nerve ECM is quite low [72]. A biomimetic scaffold that also considers the ECM characteristics improves the cellular differentiation of MSCs because of the activation of the mechanosensitive pathway [73]. The network of the scaffold must physically entrap the cells at the site, provides adhesive sites for cell survival anchorage, and elicits differentiation signals.

In all subsequent studies, three different models of SCI were used: the contusive one, which is the most used, the complete transection model, and in animals of greater size than mouse, a compression after the contusive damage was applied to better simulate the human SCI.

### 4.1. Combination of MSCs with Collagen in SCI Models

As mentioned before, collagen is an excellent material because it is biocompatible, not very antigenic, and has great mechanical properties if modified with additional materials. Different types of MSCs were used in association with collagen: BM-MSCs, placenta-derived mesenchymal stem cells (P-MSCs), and umbilical cord mesenchymal stem cells (UC-MSCs). hP-MSCs and hUC-MSCs are of human origin, and the use of xenogenetic MSCs highlights the potential for MSCs to survive, engraft, and be immunogenic in different species.

Li et al. [74], Peng et al. [75], and Liu et al. [76] used allogenic BM-MSCs. The first two used rats in acute SCI models. The third instead used a chronic canine SCI model, confirming that the collagen scaffold is a good biomaterial. Li et al. used 10^6^ BM-MSCs, and as scaffold they employed rat tail collagen I, inserting it directly into the lesion in the rat model with a completely transected spinal cord. Studies were conducted after 21 post-transplantation days. An increase in axonal growth associated with positive neurofilaments 200 (NF-200) expression was evidenced in the SCI rats transplanted with a BM-MSC-enriched scaffold. They also saw an increase in CD31, associated with vascular regeneration, and decreased stain for CD11b, a marker of macrophages, indicating a decrease in inflammation [74]. Moreover, Peng et al. found that the insertion of a collagen scaffold grafted with BM-MSCs (10^6^ cells) into a lateral hemisection of the rat spinal cord resulted in various beneficial effects. The scaffold was an aponeurosis of 0.5 mm thickness without fat and connective tissues in which cellular components and soluble proteins were extracted chemically. It was freeze-dried, sterilized, and cut into 2 mm × 2 mm × 3 mm bundles. The scaffold guarantees less diffusion of the cells with which it is enriched, keeping them at the lesion site; here, the combination induced, after 8 weeks, the regeneration of the spinal cord with greater tissue and fiber preservation in and around the scaffold, which acted as a guide thanks to the porosity of the scaffold itself. In addition, combined implantation has been reported to promote anti-inflammatory M2 macrophage polarization in situ and reduce glial scar formation and astrocyte aggregation at the injury site. All these results translated into decreased mortality and improved motor function. The model used by Peng et al. is not a canonical model, as they cut only a part of the spinal cord and not all of it [75]. Liu et al. choose the canine model (beagles) with a complete T8 section of the spinal cord and applied a 0.6–1.5 mm long and 5 mm diameter bundle of collagen scaffolds (NeuroRegen scaffold) enriched with BM-MSCs (10^6^ cells total) at the lesion site 3 months after the initial injury and after scar tissue removal. The scaffold was prepared from a bovine aponeurosis of 0.5 mm thickness separated from muscles and fats. The results showed that the dogs with the enriched scaffold achieved a better function recovery score than control group. Very few corticospinal tract and serotonin nerve fibers were found in the lesion site, but MAP2 and neuronal nuclei (NeuN) markers demonstrated the presence of neuroregeneration in the lesion area [76]. Deng et al. used hUC-MSCs in a rat and canine model, both with spinal cord transection. For rats, they used 4 mm diameter scaffolds enriched with 10^6^ cells total, while in the beagles they used scaffolds that were 5 mm in diameter and 3 mm long, enriched with 10^7^ cells total. The scaffolds were implanted immediately after the SCI induction. They observed similar results in both models. In rats, 8 weeks after injury, they noticed an improvement in locomotor function in the group with the scaffold and MSCs. Instead, in dogs they performed the Olby test and an electrophysiological examination at 3, 4, 5, and 6 months after injury. All these results showed that the implantation of the hUC-MSC-laden CS improves muscle strength and translates into more frequent weight-bearing behavior during movement compared to the control group. In the canine model, they saw, with the magnetic resonance, an increased regeneration of nerve fibers after 6 months [77]. 

Deng et al. also tried to improve the scaffold by inserting materials that increase its mechanical strength because in situ survival is closely related to scaffold mechanical stability, as the cells must adhere. The first scaffold of Deng et al. presented silk fibroin to improve the scaffold of collagen. The mass ratio was fibroin/collagen 3:7. Silk fibroin (SF) is a natural biopolymer with high biocompatibility, low immunogenicity, and sufficient biodegradability, physical strength, and flexibility. The scaffold is a blend of silk fibroin obtained from the treatment of silkworm cocoons and collagen obtained from the treatment of silver carp skins. The hUC-MSCs (100 μL of 1 × 10^5^ cellule/mL) were seeded for 7 days. The scaffolds with the cells were grafted into the rat SCI model induced by complete spinal cord transection. They observed an increased Basso Beattie Bresnahan (BBB) score, while the amplitude and latency were markedly improved in the motor-evoked potential (MEP). These tests indicated an improvement in neurological function; after 8 weeks they showed that the nerve fibers were regenerated and that myelin sheaths increased in the lesions and in the axonal number [78]. The second scaffold of Deng at al. showed improved mechanical properties thanks to heparin sulfate. Deng et al. seeded differentiated UC-MSCs, previously cultured for a week in neural differentiation medium, and then they inserted the cells and the scaffold in a canine model with spinal cord transection. The synthesis of the scaffold starts from a bovine aponeurosis without adipose tissue that was treated to obtain a purified collagen gel. The mass ratio of collagen/heparin sulfate was 20:1. Heparin sulfate is a glycosamino-glycan present in neuronal basement membrane, which plays an important role in neural regeneration and in guiding axonal regeneration. The hUC-MSCs (1 × 10^7^ cells total) and the scaffolds were co-cultured for 7 days, hUC-MSCs adhered firmly on the surface of the scaffolds, and the cells grew inside the pores. After 1, 3, and 6 months, the recovery of motor function was rated using the Olby scores, which clearly showed an improvement, and the electrophysiological study showed an increase in the amplitude and latency of the MEP. After 6 months, the magnetic resonance imaging (MRI) and diffusion tensor imaging (DTI) revealed more nerve fibers at the sites of SCI. Urodynamics was performed in all groups at 6 months, post-operatively indicating an improvement. These tests were carried out because urination is an essential outcome in the assessment of neurological recovery. The levels of IL-1β and TNF-α were suppressed after 7 and 14 days, while the levels of IL-10 and TGF-β1 significantly increased, indicating that the transplantation could upregulate the expression of anti-inflammatory factors and downregulate the expression of pro-inflammatory factors [79]. One study that confirms the Deng et al. studies of neurogenesis in acute SCI is that of Zou et al. They used a longitudinal collagen sponge scaffold enriched with hUC-MSCs (density of 2 × 10^6^ cells) grafted immediately after the completely transected spinal cord in rats. The collagen membranes were treated and lyophilized. Then, to fabricate a longitudinally oriented scaffold, they were cut into the appropriate shape and size. The scaffold was soft and molded into a cylinder shape of 2 mm in thickness and 3 mm in diameter to adapt to the lesion area of the SCI rat. Then, 8 weeks after transplantation, MEPs were examined with electrical signal transmission to assess the recovery of motor function and they saw its increase. The hMSCs efficiently reduced glial scar formation, as confirmed by the reduction in CSPG. Furthermore, there was an increased expression of NF, GFAP, GAP-43, and class III β-tubulin. These results indicated that the implantation effectively facilitated neurogenesis. Moreover, the study of inflammation showed a decrease in CD68 protein over time in the damaged zone. However, the same study also evaluated human fetal spinal-cord-derived neural stem cells, which induced a better outcome [80]. Another study that provided excellent results with hUC-MSCs in chronic SCI is that of Wang et al. This study was aimed at evaluating the effects of grafting a collagen scaffold (NeuroRegen) enriched with MSCs derived from human umbilical cord (hUC-MSCs) in the morphological and functional recovery of SCI rats undergoing surgical resection of the glial scar. The study confirmed that rats with chronic SCI had a spontaneous recovery and that it remained unchanged, even after scar removal. The scaffold synthesis procedure was the same as in Peng et al., using a 4 mm diameter collagen scaffold bundle. The implantation of the scaffold and hUC-MSCs seeded with the density of 1 × 10^6^ cells showed an increase in the BBB score compared to the other experimental groups. The 24-week follow-up confirmed persistent therapeutic effects. The experiment showed, through indicators of the electrical signal conduction capacity, that the scaffold implant combined with hUC-MSCs promoted the speed and volume of conduction of electrical signals through the chronically damaged spinal cord. The results indicated that this type of implant inhibited the process responsible for the formation of the glial scar and promoted the repair of the myelin sheath and axonal regeneration [81]. Han et al. examined the effects of 5 mm long linear-ordered collagen scaffold (LOCS) enriched with 1 × 10^7^ human placenta-derived mesenchymal stem cells (hPMSCs) transplanted in the fully dissected spinal cords of beagle dogs. Over the next 36 weeks, the scaffold-seeded transplanted dogs recorded greater motor improvements in the hind legs than the control group. The use of LOCS + hPMSCs recorded a reduction in cystic cavities and of the expression of the proteoglycans of chondroitin sulfate (CSPG). In accordance with these results, neuroregeneration was detected with class III β-tubulin and NeuN [82]. All the studies reported in this paragraph are summarized in Table 1.

### 4.2. Combination of MSCs with Fibrin in SCI Models

In all the reported studies, fibrin is presented as an excellent candidate for the creation of a scaffold with MSCs. In many cases, fibrin is used as an injection of fibrin glue due to its ease of use. In other more complex systems, cases are created in which the fibrin fibers are oriented. The most used MSCs with fibrin are BM-MSCs and AD-MSCs. 

Luzzi et al. used xenogeneic ovine 6 × 10^6^ cells/mL of BM-MSCs with a fibrin scaffold (fibrin glue produced by Tisseel, Baxter BioScience™, Deerfield, IL, USA) in a group of rats with a complete transection SCI model. They used ovine xenogeneic MSCs because they are known to migrate across the blood–brain barrier. Their study pointed out that even the xenogeneic MSCs have the potential to survive and to engraft into the injured rat spinal cord, showing signs of transdifferentiation into “neuron-like” and “glia-like” phenotypes, as demonstrated by increases in nestin, NG2, ß-III tubulin, NSE, vimentin, NF-01, and most importantly, the ability to support functional recovery (BBB score) [83]. To evaluate if MSC differentiation can ameliorate SCI, Chandrababu et al. differentiated rat AD-MSCs into neural progenitor cells (NPCs) and oligodendrocyte progenitor cells (OPCs) using a specific medium. They grew AD-MSCs in vitro on a fibrin scaffold and induced the creation of neurospheres within our progenitors that were propagated and then transplanted in the contusive lesion SCI model. Fibrin created a niche, improving AD-MSC proliferation and stable differentiation into NPCs and OPCs in vitro. Using a microliter syringe, they transplanted ∼10^4^ cells suspended in 25 µL of medium/fibrin in acute SCI. The studies clearly showed how fibrin improves SCI compared to the same cells transplanted only with the medium. Chandrababu et al. noticed severe degrees of degenerated neuronal cell bodies in all groups except the one treated with the scaffold enriched with cells where there was only a moderate loss of neurons and also a decrease in astrogliosis, cavitation, and macrophage infiltration [84]. Another study that exploited the use of AD-MSCs and showed the activation of neuroregeneration is that of Mukhamedshina et al. in which they used a rat contusive SCI model implanted with the scaffold seeded with AD-MSCs. AD-MSCs (1 × 10^6^ cells) mixed with Tissucol fibrin sealant (18 μL, Baxter) were applied on top of the injury. They saw motor recovery, using the BBB rating scale, from 1 to 11 weeks after injury, and to confirm this data an improvement in the electrophysiological studies was also found, that is, a rise in the M-wave. Furthermore, they saw a reduced cavity volume and improved tissue retention in the subacute phase following SCI. On day 60, after the application of the scaffold, they saw lower levels of GFAP and (ionized calcium-binding adapter molecule) Iba1, indicating reduced astroglial activation and a decrease in the number of microglial cells, respectively. Upregulation of the gene expression of HSPA1b and PDGFβR was found, which may be involved in neuroregeneration [85]. 

The formation of the glial scar is important in SCI because it hinders the regeneration of the spinal cord. For this reason, Garcia et al. [86], Ibarra et al. [87], and Barrera et al. [88] combined the scaffold with an injection of dipyridyl (DPY) and INDP. The first is a chelating molecule that inhibits the formation of type III collagen fibers, and consequently scar formation, while the second are neural-derived peptides with the aim of immunization. All three studies used a mixture of MSCs (2.5 × 10^6^ cells in 5 µL), and FG (Baxter^®^; 10 µL) was grafted at the site of injury.

The Garcia et al. [86] studies used an acute SCI model where 72 h after the contusive damage the necrotic tissue was removed, and the SCI rats were inoculated with the MSCs. The administration of drugs began after the insertion of the scaffold. Immunization with INDP was performed only once, while the DPY administration was performed several times in the predetermined time frame.

The combination of the treatments gave very positive results in the locomotor function thanks to a better score of BBB and a better sensibility evaluation. After 60 days, the histopathological study showed a significant increase in the amount of spared tissue and the increased axonal density in rats treated with the drugs and the fibrin scaffold enriched with MSCs (DPY + INDP + FG + MSCs) and in rats treated only with the drugs (DPY + INDP) [86]. Barrera et al. and Ibarra et al. used chronic SCI models where, 60 days after injury, they removed the scar and transplanted the scaffold with the BM-MSCs. The removal of the scar allowed the regenerating axons to grow across the site of the injury. A key difference between the two studies lies in the fact that Barrera et al. used a contusive model, while Ibarra et al. used a model with a complete spinal cord transection. The Ibarra et al. results showed increased expression of regeneration-associated genes such as NT-3, BDNF, and GAP-43. Certainly, one of the most important and evident findings was the partial recovery of the evoked potentials studying spinal cord dorsum potentials (CDPs) at the lumbar and thoracic levels in association with motor recovery [87]. In contrast, Barrera et al. noted that the best results were given by the use of the INDP alone in a chronic contusive SCI model. The combination of scaffold and MSCs showed an increase in motor recovery, but there were no increases in GAP-43, BDNF, or neuroregeneration [88].

Thanks to Mukhamedshina et al., multiple MSCs can be compared because in their study they used three types of allogeneic MSCs: BM-MSCs, AD-MSCs, and dental pulp MSCs (DP-MSCs). In addition, they did not use only rats as a model but also pigs with an induced SCI contusion. The number of cells used was based on the animal: 1 × 10^6^ cells per rat and 8 × 10^6^ cells per pig. The quantity of fibrin matrix was 18 μL for rats and 150 μL for pigs. The scaffold and MSCs were applied 2 weeks after the injury in rats and after 6 weeks in pigs. To create a man-like SCI model in the pig, they also applied pressure after the contusive damage. They studied the distribution and survival of MSCs in the rats’ lesions; after 30 days, they noticed that MSCs predominantly migrated through the posterior roots of the spinal cord, and they also saw a diminished survival of DP-MSCs. Despite the low number of cells, DP-MSCs still managed to give good results in the BBB score, while the BM-MSCs gave the worst results of the three MSCs. However, BM-MSCs and AD-MSCs gave the best indicator of MEP recovery. The best results in terms of the restoration and integrity of the tissue were given by the AD-MSCs, as confirmed by a morphometric analysis, a reduction in astroglial activation caudally, and nervous tissue regeneration. After the studies were carried out in rats, they chose to implant the scaffold enriched with AD-MSCs in pigs, obtaining results similar to those found in the rats, such as a reduction in astroglial activation caudally and nervous tissue regeneration. Despite that, the positive BBB score result was not replicated in pigs [89]. 

All the studies mentioned above used a fibrin scaffold in which the fibers were disorganized, whereas Yao et al. showed the true potential of a scaffold by using a low modulus and an aligned topography of soft aligned fibrin hydrogel scaffold (AFG). They did so because it can orient the cellular adhesion, favor neural differentiation, and induce the migration of host neural cells into the scaffold to form aligned cell fibers. To form microcell shell fibers, allogenic BM-MSCs were cultured on the micro-AFG surface, and the cells were made to adhere by holding them in suspension and rotating. The AFG was immersed into a tube containing 1 mL of cell suspension with 1 × 10^6^ cells/mL. The tube was rotated at 5 rpm in the CO_2_ cell culture incubator for 5 h, and then the AFG with the holder was extracted to culture in a growth medium for the formation of cell fibers. After 3 days of culture, the cell fibers were cut into 4 mm lengths and stacked to be implanted into the SCI lesion site. The results showed abundant NF- and GAP-43-positive nerve fibers regenerated in the caudal, rostral, and middle sites of the injury area. In addition, they showed improvements in the electrophysiological expression and limb motor functions as well as host neuron immigration into the lesion site and the neural differentiation of donor MSCs [90]. 

Spejo et al. used a fibrin seal mixing three components: fibrinogen derived from buffalo blood, 25 mM calcium chloride, and a thrombin-like protein obtained from rattlesnake venom. They saw that the combination of BM-MSCs and fibrin sealant in a scaffold had better effects than individual ones in motor neuron survival rate and M2 macrophage concentration, although considering the change in cytokine concentration, the combination led to a pro-inflammatory profile. This could be justified by the model used for creating SCI, which was completely different from the contusive or complete transection models that are used in rats. Spejo et al. used as a model a unilateral ventral funiculus cut, leading to an intraspinal axotomy of motor neurons at spinal levels L4, L5, and L6 in rats [91]. All the studies reported in this paragraph are summarized in Table 2.

### 4.3. Combination of MSCs with Chitosan in SCI Models

Chitosan can be used as a hydrogel or nanofiber scaffold. As a hydrogel, it has particularly interesting features, such as being thermosensitive and being a liquid when outside the body, In fact, it becomes a hydrogel only once it is injected. This makes it easier to use in case of SCI and increases the number of cells that can be loaded.

Basak et al. gave chitosan the shape of a tube, seeded it with allogenic BM-MSCs, and evaluated its effects in rats with a complete spinal cord transection model. The tubes were 10 mm in length and 4.1 mm in outer diameter, and the wall thickness was 0.21 mm. The BM-MSCs were stuffed into the chitosan channels at a density of 0.5 × 10^6^/10 mL of complete medium. The MSC application slightly accelerated the spinal cord and myelin sheath repair processes. The BBB scores showed no significant improvements [92].

Zhang et al. used an injectable thermosensitive scaffold with BM-MSCs in mice with contusive SCIs and saw that the scaffold was highly biocompatible and not inflammatory. The total cell number in the BM-MSCs group or the scaffold+BM-MSCs group was 1 × 10^6^ per mouse. An important result they obtained was the improvement in the low mouse scale test after 28 days, which indicated that the recovery of hindlimb motor function in mice treated with scaffold+BM-MSCs was the best. They also evaluated the emotional state of SCI mice on day 28 after treatment using the tail suspension test and the sucrose preference test. In these tests, we can also see marked improvements with scaffolds and BM-MSCs. Moreover, in this study, a large decrease in edema was found. Lastly, increased protein expression of MAP2, enolase 2, and the neuronal migration protein doublecortin was observed, suggesting the better survival of neurons and neurogenesis. In association, a reduction in Bax and Bcl-2 was observed, indicating the enhanced suppression apoptosis. Furthermore, the expression of BDNF and neurotrophin-3 (NT-3) was higher, indicating the enrichment of neurotrophic factors [93].

Zhang et al. [93] proved once again that BM-MSCs have potential neurogenic cell differentiation capacity and the ability to secrete numerous neurotrophic factors or growth factors that are important for neuronal survival and differentiation. 

Given the potential of BDNF and NT-3, Ji et al. used AD-MSCs overexpressing BDNF and NT-3, in association with a chitosan scaffold created by combining fibroin silk and chitosan, to increase the mechanical strength and water absorption. A 200,000-cell suspension was added to the scaffolds of 2 mm × 2 mm dimensions and left for 4 h. The cell medium was changed every 3 days from 10 days after the inoculation in preparation for the in vivo animal experiment. They saw that AD-MSCs overexpressing BDNF-NT3 combined with the scaffold improved motor function in the SCI model. After 12 weeks, an improvement in the spinal cord morphology, a reduction in scar tissue, and a better anastomosis between the spinal cord and the scaffold were observed. There was no obvious inflammatory reaction between the scaffold and the spinal cord, but many neuron-like cells were observed inside the scaffold. In the BDNF-NT3 group, what caught the attention was a large amount of nerve fibers that were fully formed, morphologically normal, and consistent in shape. In addition to the marked morphological and functional improvement, they observed an increase in the expression of GAP-43 and reductions in GFAP and Casp-3. The inhibition of apoptosis can prevent or reduce secondary injuries, protect nerve function, and relieve nerve cell loss [94]. 

Alizadeh et al. evaluated the potential of NGF-overexpressing AD-MSCs in association with injectable chitosan/ß-glycerophosphate/hydroxyethylcellulose hydrogel for SCI treatment. The CS/β-GP/HEC hydrogel had a porous structure and showed the high surface area and high porosity with uniform interconnected pores ranging from 100 to 150 μm. NGF has been seen to play a fundamental role in neuronal development and axonal remodeling as well as axon regeneration after SCI, but the half-life does not allow its proper use. Therefore, the application of NGF-overexpressing AD-MSCs with a scaffold may associate the positive actions of NGF and AD-MSCs, increasing and prolonging their effects. This thermosensitive scaffold was injected a week after the contusive SCI model induction. They saw increased repair during the 8 weeks postinjection, as confirmed by the production of ECM components such as lamina and more transfected cells in the lesion with the scaffold [95]. To improve the neuroregenerative ability of MSCs, Huang et al. transfected BM-MSCs with an adenovirus containing the glial-cell-derived neurotrophic factor (GDNF) gene, differentiating them from neural-like cells. In vitro, the transfected cells cultured with the scaffold showed a better bipolar or multipolar synaptic structure of neural-like cells and enhanced differentiation into mature neurons and astrocytes compared to the same cells without the scaffold. In vivo, an increase in the markers of neural differentiation and glia, such as NeuN, NF-200, and GFAP was observed. Moreover, a decrease in CSPG, known to inhibit axon regeneration after SCI, was found. The results indicated that the rats implanted with the scaffold and transfected BM-MSCs showed an earlier functional improvement and continually displayed higher scores of locomotor function from weeks 1 to 5 after surgery. More interestingly, immunofluorescence showed more interconnected and orderly arranged axons in the lesion site 6 weeks after transplantation [96]. The study conducted by Boido et al. used a chitosan scaffold and did not focus so much on MSCs but on their secretome and their paracrine potential. The porous structure of the scaffolds showed two-scale porosity: a macroporosity with a pore size around 112 ± 23 µm and a microporosity with a pore size around 15 ± 7 µm. A total of 150,000 MSCs were embedded in CS/β-GP hydrogel and injected immediately after murine SCI transection. In this way, they showed how the secretome of MSCs combined with chitosan can reduce inflammation and does not promote the creation of the glial scar [97]. 

Song et al. tried a combined approach using a scaffold that released the drugs in two months. To do this, they used the combination of electrospinning and electrospray to make a membrane composed of polylactic acid (PLA), PLGA, and chitosan. The electrospray technique transmitted a high voltage to a polymeric solution to force the polymer to come out from a nozzle and take the form of nano/microspheres. PLA was obtained by electrospinning and had the function of mechanical support and sealant to avoid drug diffusion. PLGA was used to make microspheres (6–11 µm) that released NGF in a controlled manner and were applied to the PLA layer by electrospray. By electrospinning, a CS state was applied to serve as a seeding layer for BM-MSCs seeded with the density of 2 × 10^6^ cells. The inclusion of NGF had the aim to promote the growth and differentiation of nerve cells for SCI therapy. The direct long-term administration of NGF had some problems, such as being easily degradable and difficult to pass through the blood–spine barrier and the increased risk to generate tumors. The scaffold with the dimensions 5 mm × 8 mm was implanted into rats in a contusive SCI model. The results of this work demonstrated that NGF/BM-MSCs combined with the scaffold resulted in a marked functional motor recovery and increased the expression of GAP-43 and NF-200, indicating neural regeneration [98]. All the studies reported in this paragraph are summarized in Table 3.

### 4.4. Combination of MSCs with PLGA in SCI Models

In the reported studies, PLGA was implanted as a scaffold in rat models with SCI. It was effective in filling the gap caused by the lesion and was not cytotoxic for MSCs. In addition, this biomaterial has been used to create nanoparticles capable of carrying growth factors and neutrophils. These studies demonstrate that PLGA is a suitable and versatile biomaterial in the field of neurogeneration. 

The study by Xu et al. used rats with hemilesions between T9 and T11 as an experimental model and divided them into four groups: (1) a control group (rats with SCI), (2) a BBC group in which they implanted a scaffold consisting of acellular spinal cord (ASCS) with empty PLGA nano particles (B-ASCS) and BM-MSCs into the lesion, (3) a VNA group in which they implanted ASCS with PLGA nanoparticles with NT-3 and PLGA nanoparticles with VEGF (VN-ASCS) into the lesion, and (4) a VNBC group in which rats with SCI were implanted with a construct of VN-ASCS/BM-MSCs. The dimensions of the scaffolds were 3 mm long and 2 mm in diameter, and the number of the cells was equal to 30 μL of 1 × 10^6^ cells/mL cell suspension. This work showed that the aforementioned factors are released by the nanoparticles at the lesion site and lead to a reduction in inflammation, an increase in angiogenesis, and a decrease in glial reactivity, limiting the formation of a glial scar. In addition, the factors promoted axonal regeneration. Enrichment with BM-MSCs of the ASCS/VN-NP reduced the macrophage concentration and improved the axonal regeneration, resulting in a significant motor recovery in rats [99]. The study of Alexander E. Ropper et al. implanted PLGA scaffolds enriched with human-derived BM-MSCs into the T9-T10 hemitransection site in injured rats. The scaffold was cut with the dimensions of 1 mm × 2 mm × 4 mm for in vivo implantation and with pore size diameters of 350–500 μm. The number of hMSCs incorporated in the scaffolds was ∼5.0 × 10^5^. The results demonstrated that the presence of the seeded scaffold prevents neuropathic pain, limits demyelination, promotes neurogenesis, promotes M2 macrophage polarization, and downregulates pro-inflammatory cytokines, T cells, and M1 macrophages. This work clearly demonstrates the motor recovery of SCI rats after MSC-enriched scaffold implantation. Furthermore, it emphasizes the importance of the stiffness and three-dimensionality of the enriched scaffold to avoid the undesirable differentiation of hMSCs into mesenchymal tissues [100]. The study by Yang et al. applied a PLGA scaffold with 50 microchannels and a rod shape 5 cm in length and 3 mm in diameter enriched with rat BM-MSCs. A volume of 10 μL of cell suspension (1 × 10^7^ cells/mL) was added per scaffold. In this case, the applied enriched scaffold eliminated the gap created by the lesion and directed nerve regeneration along the injured medulla. Unlike the previous work, the rats underwent a complete transection of the spinal cord, reaching the BBB value of zero. The application of the enriched scaffold, as in the previous work, favored nerve regeneration, presumably allowing motor recovery. In addition, this study found a reduction in the cystic area and an improvement in the MEP and the somatosensory evoked potential (SEP) [101]. The study by Han et al., as in that of Alexander E. Ropper et al., used rats with thoracic spinal cord hemisection, and the PLGA scaffold used was enriched with human-derived BM-MSCs. This last work showed that the enriched PLGA scaffold promoted, according to previous works, a reduction in inflammation and motor recovery of rats but only if the scaffold had a PLGA concentration of 10% in the solvent during scaffold casting in the molds (soft scaffold), as a higher concentration reduced the beneficial effects. The scaffolds had pore sizes of 350–500 μm and were cut to the implant size of W × H × L: 1 mm × 2 mm × 4 mm. The work also found reduced white matter tissue loss and the increased protection of interneurons. In light of these results, the use of PLGA in SCI can be useful in the transport of factors or even to constitute the actual scaffold but with the condition that it has a consistency and architecture that allows the right nervous growth [102]. All the studies reported in this paragraph are summarized in Table 4. 

**Figure 1:**
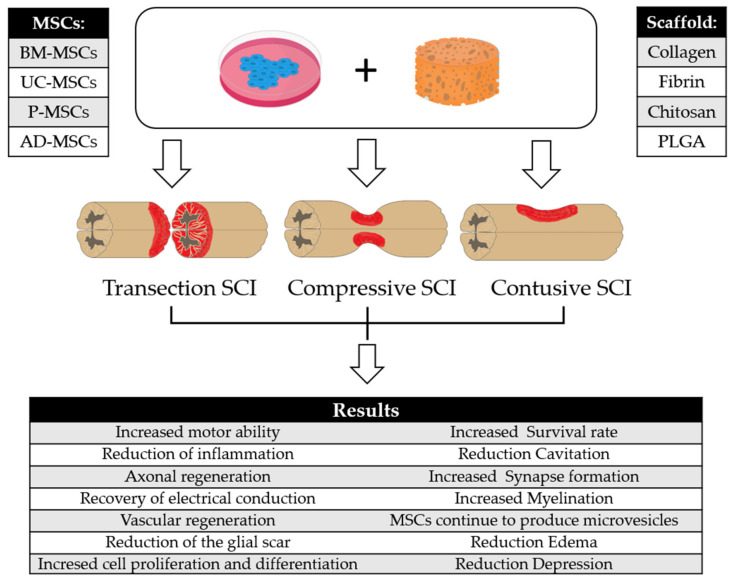
Results obtained using different types of MSCs combined with different biomaterials in the three models of SCI. Figure drawn using the vector image bank of Servier Medical Art by Servier (http://smart.servier.com/, accessed on 27 June 2022). Licensed under a Creative Commons Attribution 3.0 Unported License (https://creativecommons.org/licenses/by/3.0/, accessed on 27 June 2022).

Figure 1 is a summary of the results obtained in different studies with the scaffolds and MSCs.

## 5. Clinical Studies with Scaffold Enriched with MSCs for Treatment of Spinal Cord Injury

In the clinical trial NCT02352077 by Yannan Zhao et al., they recruited eight patients with complete SCI with a mean duration of SCI of approximately 13.4 months at the thoracic or cervical level. In these patients, NeuroRegen scaffolds, biodegradable collagen scaffolds enriched with hUCB-MSC, were implanted at the site of injury after the removal of the glial scar. Patients underwent motor and sensory rehabilitation for 6 months after enriched scaffold implantation. The results demonstrated improvements in the level of sensation in five patients, increases in the MEP reactive area in seven patients, increased trunk stability in four patients, and recovery of defecation sensation in two patients. No adverse events were observed during the follow-up period. According to the study, these findings can be attributed to the regeneration of ascending axons. In addition, they showed increased sweating below the level of injury, indicating partial recovery of the autonomic nervous system [103]. 

In the study by Zhifeng Xiao et al., two patients were recruited, including one with complete thoracic SCI and one with complete cervical SCI (NCT02510365). The NeuroRegen scaffold enriched with hUCB-MSC was grafted into these patients. The enriched scaffold was transplanted approximately 24 h after injury in the patient with thoracic SCI, whereas the transplantation occurred 8 days after injury in the patient with cervical SCI. The authors used the Walk Index for SCI (WISCI) to assess the patients’ motor skills and evaluated the patients at regular intervals for 1 year. The results showed that within 1 year after surgery the patient with thoracic SCI gradually recovered the ability to walk. At 3 months after surgery, the patient experienced improvement in adductor magnus contraction, and at 6 months they experienced improvements in the motor mobility of the lower limbs and toes. With the SEP and MEP parameters, the authors demonstrated that the patient showed a recovery of electrical conduction in the spinal cord. The patient with cervical SCI began to show motor and sensory recovery two months after surgery. At 12 months, the patient recovered sensory function of the bowel and bladder. At a 6-month follow-up, the patient was able to lift his lower limbs against gravity and move his toes. The SEP and MEP parameters confirmed a partial recovery of spinal cord nerve transmission [104].

In the study by Fengwu Tang et al., patients with acute and chronic SCI were recruited, and NeuroRegen scaffolds enriched with mononuclear cells from the patients’ own bone marrow (BMMNCs) or human umbilical cord mesenchymal stem cells (UCB-MSCs) were implanted (NCT02510365 and NCT02352077). A total of 15 patients with acute SCI with ages ranging from 22 to 60 years were enrolled. Six patients had cervical lesions, and nine had thoracic lesions. Five patients underwent implantation of the NeuroRegen scaffold enriched with patients’ BMMNCs, whereas seven patients received the NeuroRegen scaffold enriched with UCB-MSCs, while three patients received an implantation of the NeuroRegen scaffold alone. In parallel, 51 patients with chronic SCI with a mean duration of SCI of 21.69 months (range, 2–80 months) were recruited. The patients’ ages ranged from 19 to 61 years. A total of 16 patients had SCI in the cervical segments, and 35 had lesions in the thoracic segments. Of the 51 patients, 22 patients underwent implantation of an UCB-MSC-enriched NeuroRegen scaffold, whereas 29 patients underwent implantation of a combined NeuroRegen scaffold with BMMNCs. Four patients with acute SCI recovered their ability to walk. Of these patients, two had a cervical lesion and began to recover walking ability 1 year after surgery. The walking index for SCI (WISCI) was applied as a motor assessment parameter. In this case, the two patients had an improvement in the WISCI parameter from 0 to 6 and from 0 to 9. The other two patients who demonstrated motor recovery had a thoracic injury. One thoracic injury patient with motor recovery began walking with a supportive brace 6 months after surgery, with a gradual increase in WISCI from 0 to 9 with a 12-month follow-up. To determine the neural conductivity in patients, SEP and MEP parameters were used, showing that patients who regained the ability to walk also had improvements in neural conductivity. Three patients regained bowel and bladder sensation. The results reported for patients with chronic SCI showed that 31.37% of patients had an improvement in sensation level. Seven of the sixteen patients with cervical lesions had an increase in finger flexibility or shoulder activity. In contrast, patients with thoracic lesions had no motor improvement, but 75% experienced expansion of MEP response positions. In addition, 58.82% of patients partially recovered defecation sensation, and 58.82% showed increased sweating of the skin under the injury sites. The study showed that the greatest improvements occur within two years after the graft of the enriched scaffold [105]. These three studies demonstrate that the application of the NeuroRegen scaffold enriched primarily with hUCB-MSCs in lesions of patients with complete SCI can create a favorable microenvironment for nerve regeneration and motor recovery, especially in the first 2 years of follow-up, and that these applications may be useful in creating therapy for acute and chronic SCIs.

## 6. Future Perspectives

The results showed in the studies are promising. The main problem is represented by the absence of standardization since the use of MSCs and the creation of scaffolds vary according to the protocol used in each laboratory. One solution could be the use of standardized biomaterial, which can be provided by specialized companies. An extremely promising strategy to treat SCI is to differentiate MSCs or to make them overexpress neuroprotein in vitro before transplanting them in vivo. One of the reasons why MSCs are used is because they can differentiate in situ, so the pre-treatment can be a better way to increase the number of cells that can heal the SCI. The results obtained from the experiments conducted in pig and dog models, which could simulate human SCI well, are encouraging. As are the results from the clinical trials. The evidence suggest that more clinical trials should be conducted.

Further studies are also needed to clarify the molecular mechanisms of mechanotransduction to understand how cells are affected in their differentiation inside a 3D model and to ameliorate the MSC–scaffold interaction and their therapeutic effects. The scaffold enriched with MSCs can also be improved in order to simulate the staminal niche and, in this way, promote healing by also performing stem cell homing at the site of damage. In order to improve the use of drugs or growth factors when the scaffold is implanted, the combination drug scaffolds must be increased, or the drugs could be directed to the scaffolds after the infusion using specific antibodies. 

## 7. Conclusions

The combination of MSCs and scaffolds shows beneficial results in almost all studies, using both allogenic and xenogeneic MSCs. However, collagen is the most used biomaterial in vivo and in clinical trials thanks to its biocompatibility and absence of immunogenicity. Fibrin was used in numerous in vivo studies with excellent results in both chronic and acute SCI. Chitosan, with its positive charge and the possibility to create hybrid scaffolds, proved to be an extremely promising biomaterial. PLGA is widely used to perform drug delivery, so there are many studies on its safety. This material improves its performance in combination with other biomaterials for drug delivery purposes.

The results highlighted the ability of MSCs to differentiate as well as that the secretome improves inflammation. It is also clear that the scaffold features must be optimized to promote neuroregeneration because the fate of stem cells is also regulated by biophysical cues. Moreover, the use of drugs together with scaffolds and MSCs greatly improved the damaged area. A concern for the study of SCI is that in vitro models are not complete enough, and in vivo models are needed, but they are very complex to standardize. In addition, the administration of cells in the acute or chronic phases completely changes the obtained results. Indeed, the models are obtained through different types of lesions, and the timing of scaffold transplantation and analysis can vary. These important differences create several difficulties in comparing the results.

## Figures and Tables

**Table 1:** Overview of the studies evaluating biochemical and cellular changes using MSC-enriched collagen scaffolds in in vivo SCI models.

MSCs	Scaffold	Model	Results	Reference
Allogenic BM-MSCs 1 × 10^6^ cells total	7 µL of rat tail collagen I injection	Rats: completely transected spinal cord Analysis was conducted after 21 days	↑Axonal regeneration ↑Vascular regeneration ↓Inflammation	[74]
Allogenic BM-MSCs 1 × 10^6^ cells total	Porous collagen scaffold with the size of 2 × 2 × 3 mm	Lateral hemisection SCI rat model	↑Survival rate ↑Motor recovery ↓Glial scar formation	[75]
hBM-MSCs 1 × 10^6^ cells total	Collagen scaffold with the dimensions of 0.6–1.5 mm long and 5 mm diameter	Completely transected spinal cord in beagles	↑Neurogenesis ↑Locomotor recovery	[76]
hUC-MSCs 1 × 10^6^ cells total in rats 1 × 10^7^ cells total in beagles	Collagen scaffold 4 mm diameter in rats, 5 mm diameter and 3 mm long in beagle	Completely transected spinal cord in rats and beagles	↓Lesion area ↑ regeneration of nerve fibers ↑Neurological function	[77]
hUC-MSCs 1 × 10^6^ cells total	Silk fibroin/collagen with mass ratio of 3:7 scaffolds with the dimension of 2 mm	Rats: complete spinal cord transection Analysis was conducted after 8 weeks	↑Axonal regeneration ↑Myelination ↑Locomotion recovery	[78]
hUC-MSCs incubated for 1 week in neural differentiation medium 1 × 10^7^ cells total	Collagen/heparin sulphate scaffold with mass ratio of 20:1	12 Beagle dogs with spinal cord transections	↑Locomotion recovery ↑MEP ↑Neurological recovery ↑Nerve fibers ↑IL-10 and TGF-β1 ↓IL-1β and TNF-α	[79]
hUC-MSCs 2 × 10^6^ cells total	Longitudinal collagen sponge scaffolds 2 mm in thickness and 3 mm in diameter	Rats: complete spinal cord transection Analysis was conducted after 8 weeks	↑Motor function ↑NF, GFAP, GAP-43, and class III β-tubulin ↓CSPG ↓CD 68	[80]
hUC-MSCs 1 × 10^6^ cells total	Collagen scaffold 4 mm diameter hydrated scaffold bundle	Rats with chronic spinal cord injury Surgical resection of the glial scar	↑Persistent motor recovery ↑Electrical conduction ↑Myelin sheath ↓Glial scar formation	[81]
hP-MSCs 1 × 10^7^ cells total	Linear-ordered collagen scaffold 5 mm long	Beagle dogs with T8 completely transected spinal cord	↑Motor recovery ↓Chondroitin sulfate proteoglycans ↑Axonal regeneration ↑Remyelination ↑Synapse formation	[82]

MSCs: Mesenchymal stem cells; SCI: Spinal cord injury; BM-MSCs: Bone-marrow-derived MSCs; ↑: Increase; ↓: Decrease; hUC-MSCs: Human umbilical cord mesenchymal stem cells; MEP: Motor-evoked potential; IL-10: Interleukin 10; TGF-β1: Transforming growth factor beta 1; IL-1β: Interleukin 1 beta; TNF-α: Tumor necrosis factor alpha; NF: Nerve fibers; GFAP: Glial fibrillary acidic protein; GAP-43: Growth-associated protein 43; CSPG: Chondroitin sulfate proteoglycans; CD68: Cluster of differentiation 68; hP-MSCs: Human placenta-derived mesenchymal stem cells; hBM-MSCs: Human bone-marrow-derived MSCs.

**Table 2:** Overview of the studies evaluating biochemical and cellular changes using MSC-enriched fibrin scaffolds in in vivo models.

MSCs	Scaffold	Model	Results	Reference
Sheep BM-MSCs 6 × 10^6^ cells/mL	Fibrin glue injection	Rats with complete spinal cord transection MSCs with fibrin were applied immediately upon transection. Rats were sacrificed after 70 days of treatment.	↑Locomotor recovery Xenogeneic MSCs showed the expression of early “neuro-like” and “glia-like” differentiation patterns.	[83]
AD-MSCs differentiated in vitro toward NPCs and OPCs ∼10^4^ cells suspended in 25 microliters of medium/fibrin	Fibrin glue injection 25 microliters of medium/fibrin	Contusive SCI model in rats Analyses were conducted 28 days after the damage and insertion of the scaffold.	↑Locomotor recovery only in control with just fibrin ↓Loss of neurons ↓Astrogliosis ↓Cavitation ↓Macrophage infiltration	[84]
Allogeneic AD-MSCs 1 × 10 ^6^ cells totals	Fibrin glue injection 18 μL of Baxter	Rats: contusion at Th8 The scaffold was applied two weeks after the SCI and analyzed after 74 days.	↑Locomotor recovery ↑Tissue retention ↑Cavity volume in the subacute phase ↑H/M wave amplitude ratio ↑neurogenesis ↓Astroglial activation	[85]
Allogeneic BM-MSCs 2.5 × 10^6^ cells in 5 µL	Fibrin glue injection 10 μL of Baxter	Contusive SCI model in rats with injection of DPY and INDP The scaffold was transplanted 72 h after the damage, and the necrotic tissue was removed.	↑Mechanical withdrawal and locomotor recovery ↑Axonal fibers ↑Motor and sensory recovery in animals treated with DPY + INDP + FG + MSCs	[86]
Allogeneic BM-MSCs 2.5 × 10^6^ cells in 5 µL	Fibrin glue injection 10 μL of Baxter	Complete spinal cord transection rats with injection of DPY and INDP The scaffold was implanted 60 days after the damage with surgical removal and inhibition of the glial scar.	↑Locomotor recovery ↑Neuron fibers and the recovery of electric activity ↑BDNF, NT3, GAP-43, and NGF	[87]
Allogeneic BM-MSCs 2.5 × 10^6^ cells in 5 µL	Fibrin glue injection 10 μL of Baxter	Contusive rat SCI model with injection of DPY and INDP The scaffold was implanted 60 days after the damage with surgical removal and inhibition of the glial scar.	↑Motor recovery ↓GAP-43 and BDNF ↓Neuroregeneration	[88]
Allogeneic BM-MSCs, AD-MSCs, and DP-MSCs 1 × 10^6^ cells per rat 8 × 10^6^ cells per pig	Fibrin Matrix FM Tissucol (18 μL) for rats FM Tissucol (150 μL) for pigs	Rat: the spinal cord contusion model The scaffolds were applied 2 weeks after injury. Pigs: compression was carried out in addition to contusion. The scaffolds were applied 6 weeks after injury.	In rats: ↑motor activity ↑neural tissue integrity ↑conduction along spinal cord ↓cavitation In pigs: ↑ neural tissue integrity ↓cavitation Partial restoration of the somatosensory spinal pathways No effect of AD-MSCs on microglia No significant improvement in motor activity scores in pigs	[89]
Allogenic BM-MSCs 1 × 10^6^ cells	Fibrin hydrogel with an AFG 4 mm in length	Rats with complete spinal cord transection	↑Regeneration of NF- or GAP-43-positive nerve fibers in the caudal, rostral, and middle sites of the injury area ↑Electrophysiological expression and limb motor functions, host neuron immigration, and neural differentiation of donor MSCs	[90]
Allogeneic BM-MSCs 1 × 10^6^ cells	Fibrin sealant	Rats with unilateral cut of the ventral funiculus	MSC therapy is neuroprotective and, when combined with FS, shifts the immune response to a pro-inflammatory profile. ↑Neuronal survival ↓Astrogliosis ↓Synaptic preservation	[91]

MSCs: Mesenchymal stem cells; BM-MSCs: Bone-marrow-derived MSCs; SCI: spinal cord injury; ↑: increase; AD-MSCs: Adipose-derived MSCs; NPCs: neural progenitor cells; OPCs: oligodendrocyte progenitor cells; Fib: Fibrin; ↓: decrease; H/M: wave amplitude ratio; DPY: dipyridyl; INDP: Immunization with neural-derived peptides; FG: fibrin glue; BDNF: brain-derived neurotrophic factor; GAP-43: Growth-associated protein 43; NGF: Nerve growth factor; DP-MSCs: dental pulp MSCs; AFG: aligned fibrillar structure; FS: Fibrin sealant.

**Table 3:** Overview of the studies evaluating biochemical and cellular changes using MSC-enriched chitosan scaffolds in in vivo SCI models.

MSCs	Scaffold	Model	Results	Reference
Allogeneic BM-MSCs The BM-MSCs were stuffed into the chitosan channels at a density of 0.5 × 10^6^/10 mL.	Tubular forming of chitosan The tubes were 10 mm in length, 4.1 mm in outer diameter, and the wall thickness was 0.21 mm.	Rats with spinal cord transection	No significant changes in BBB score ↑Axons per unit area ↑Myelin sheath repair	[92]
Allogeneic BM-MSCs 1 × 10^6^ per mouse	Thermosensitive composite hydrogel based on chitosan, hydroxyethyl cellulose, collagen, and β-phosphoglycerate	Contusion SCI mice model Analysis was conducted after 28 days.	↑Locomotor recovery ↓Depression ↓Edema ↑Survival of neurons ↑Neurogenesis ↓Apoptosis ↑Neurotrophic factors	[93]
AD-MSCs overexpressing brain-derived neurotrophic factor (BDNF) and neurotrophin-3 (NT-3) 200,000-cell suspension	Silk fibroin/chitosan scaffold with the dimensions of 2 mm × 2 mm	Rats with spinal cord transection Rats were sacrificed after 12 weeks of treatment.	↑Locomotor recovery ↓Scar tissues ↑Neuron-like cells ↓Inflammatory cells ↑GAP-43↓GFAP↓CASP-3	[94]
AD-MSCs transfected with lentiviral mediated nerve growth factor 1 × 10^5^ cells	Injectable thermosensitive hydrogel chitosan/β-glycerophosphate/hydroxyethyl cellulose Pores ranging from 100–150 μm	The scaffold was applied in rats one week after the contusive SCI induction, and the evaluations were performed after two months.	↑Locomotor recovery ↑Cell proliferation ↓Cavitation ↑Spinal cord ECM	[95]
Allogeneic BM-MSCs transfected with an adenovirus containing the glial-cell-derived neurotrophic factor gene 2 × 10^5^ cells/10 μL	Thermosensitive quaternary ammonium chloride chitosan/ß-glycerophosphate hydrogel The multiporous three-dimensional structure of the hydrogel scaffolds had an average pore size of 118.56 ± 11.92 μm.	Contusive SCI model in rats Rats were sacrificed after 2, 4, and 6 weeks of treatment.	↑NeuN, NF-200, and GFAP ↓CSPG ↑Locomotor ↑Neurogenesis ↓Cavitation Increased interconnected and orderly arranged axons in the lesion site 6 weeks after transplantation	[96]
BM-MSCs 150,000 cells	7 µL of chitosan-based hydrogel with β-Glycerol phosphate disodium MSCs were mixed with the hydrogel solution prior to gelation.	Mice with complete spinal cord transection	MSCs continued to produce microvesicles, even with the scaffold ↓ROS level reduction	[97]
BM-MSCs 2 × 10^6^ cells	PLA/NGF-PLGA/CS composite membrane The size of the composite membranes used was 5 mm × 8 mm.	Contusion SCI Rats	↑Neurogenesis ↑Locomotor recovery	[98]

MSCs: Mesenchymal stem cells; SCI: Spinal cord injury; BM-MSCs: Bone-marrow-derived MSCs; ↑: Increase; BBB: Basso, Beattie, Bresnahan; ↓: Decrease; AD-MSCs: Adipose-derived MSCs; BDNF: Brain-derived neurotrophic factor; NT-3: Neurotrophin-3; GAP-43: Growth-associated protein 43; GFAP: Glial fibrillary acidic protein; CASP-3: Caspase 3; ECM: Extracellular matrix; NeuN: Neuronal nuclei; NF-200: Neurofilament 200; CSPG: Chondroitin sulfate proteoglycans; ROS: Reactive oxygen species; PLA: Polylactic acid; NGF: Nerve growth factor; PLGA: Poly(lactic-co-glycolic acid); CS: Chitosan.

**Table 4:** Overview of the studies evaluating biochemical and cellular changes using MSC-enriched PLGA scaffolds in in vivo SCI models.

MSCs	Scaffold	Model	Results	Reference
Allogenic BM-MSCs 30 μL of cell suspension 1 × 10^6^ cells/mL	Acellular spinal cord scaffold + PLGA nanoparticles with VEGF and NT-3 Dimensions of the scaffolds: 3 mm long and 2 mm in diameter	Rats with unilateral hemisection T9 to T11	↑Motor recovery ↑Axonal regeneration ↓Macrophage infiltration	[99]
hBM-MSCs ∼5 × 10^5^	PLGA scaffolds tailored to be unique, porous, soft, and smooth dimensions of 1 × 2 × 4 mm	Rats with hemisection T9 to T10	↑Motor recovery ↑Axonal regeneration ↑Angiogenesis ↓Neural inflammation ↓Loss of tissue	[100]
Allogenic BM-MSCs 10 μL of cell suspension 1 × 10^7^ cells/mL	PLGA scaffold with 50 microchannels in rod shape of 5 cm in length and 3 mm in diameter	Rats with complete transection of the thoracic spinal cord	↑Nerve regeneration ↑Motor-evoked potential ↑Somatosensory evoked potential ↑Motor recovery ↓ Cystic area Combination with Schwann cell increased these values and promoted the differentiation of MSCs into neuron-like cells	[101]
hBM-MSCs ~6 × 10^4^ cells were seeded in the soft scaffold	Soft PLGA scaffold with pore sizes of 350–500 μm. Size of W × H × L: 1 mm × 2 mm × 4 mm	Rats with unilateral hemisection of the midline at the T9-T10 level	↑Functional recovery ↑Interneuron protection ↓ Loss of tissue ↓Loss of white matter ↓Neural inflammation	[102]

MSCs: Mesenchymal stem cells; SCI: Spinal cord injury; PLGA: Poly (lactic-co-glycolic acid); BM-MSCs: Bone marrow-derived MSCs; VEGF: Vascular endothelial growth factor; NT-3: Neurotrophin-3; ↑: Increase; ↓: Decrease; hBM-MSCs: Human bone-marrow-derived MSCs.

## Data Availability

No new data were created or analyzed in this study. Data sharing is not applicable for this article.

## References

[1] Anjum A., Yazid M.D., Daud M.F., Idris J., Ng A.M.H., Naicker A.S., Ismail O.H.R., Kumar R.K.A., Lokanathan Y. (2020). Spinal Cord Injury: Pathophysiology, Multimolecular Interactions, and Underlying Recovery Mechanisms. Int. J. Mol. Sci..

[2] Ahuja C.S., Wilson J.R., Nori S., Kotter M.R.N., Druschel C., Curt A., Fehlings M.G. (2017). Traumatic spinal cord injury. Nat. Rev. Dis. Primers.

[3] Müller-Jensen L., Ploner C.J., Kroneberg D., Schmidt W.U. (2021). Clinical Presentation and Causes of Non-traumatic Spinal Cord Injury: An Observational Study in Emergency Patients. Front. Neurol..

[4] Clark J.M., Marshall R. (2017). Nature of the Non-traumatic Spinal Cord Injury Literature: A Systematic Review. Top. Spinal Cord Inj. Rehabil..

[5] Grumbles R.M., Thomas C.K. (2017). Motoneuron Death after Human Spinal Cord Injury. J. Neurotrauma.

[6] Bains M., Hall E.D. (2012). Antioxidant therapies in traumatic brain and spinal cord injury. Biochim. Biophys. Acta (BBA) Mol. Basis Dis..

[7] Harry G.J., Kraft A.D. (2008). Neuroinflammation and microglia: Considerations and approaches for neurotoxicity assessment. Expert Opin. Drug Metab. Toxicol..

[8] Cregg J.M., DePaul M.A., Filous A.R., Lang B.T., Tran A., Silver J. (2014). Functional regeneration beyond the glial scar. Exp. Neurol..

[9] Fan B., Wei Z., Yao X., Shi G., Cheng X., Zhou X., Zhou H., Ning G., Kong X., Feng S. (2018). Microenvironment Imbalance of Spinal Cord Injury. Cell Transplant..

[10] Zhang Y., Al Mamun A., Yuan Y., Lu Q., Xiong J., Yang S., Wu C., Wu Y., Wang J. (2021). Acute spinal cord injury: Pathophysiology and pharmacological intervention (Review). Mol. Med. Rep..

[11] Stein D.M., Sheth K.N. (2015). Management of Acute Spinal Cord Injury. Contin. Lifelong Learn. Neurol..

[12] Rath N., Balain B. (2017). Spinal cord injury—The role of surgical treatment for neurological improvement. J. Clin. Orthop. Trauma.

[13] Oshigiri T., Sasaki T., Sasaki M., Kataoka-Sasaki Y., Nakazaki M., Oka S., Morita T., Hirota R., Yoshimoto M., Yamashita T. (2019). Intravenous Infusion of Mesenchymal Stem Cells Alters Motor Cortex Gene Expression in a Rat Model of Acute Spinal Cord Injury. J. Neurotrauma.

[14] Prockop D.J., Youn Oh J. (2012). Mesenchymal Stem/Stromal Cells (MSCs): Role as Guardians of Inflammation. Mol. Ther..

[15] Liu H., Honmou O., Harada K., Nakamura K., Houkin K., Hamada H., Kocsis J.D. (2006). Neuroprotection by PlGF gene-modified human mesenchymal stem cells after cerebral ischaemia. Brain.

[16] Sasaki Y., Sasaki M., Kataoka-Sasaki Y., Nakazaki M., Nagahama H., Suzuki J., Tateyama D., Oka S., Namioka T., Namioka A. (2016). Synergic Effects of Rehabilitation and Intravenous Infusion of Mesenchymal Stem Cells after Stroke in Rats. Phys. Ther..

[17] Steward O., Sharp K.G., Yee K.M., Hatch M.N., Bonner J.F. (2014). Characterization of Ectopic Colonies That Form in Widespread Areas of the Nervous System with Neural Stem Cell Transplants into the Site of a Severe Spinal Cord Injury. J. Neurosci..

[18] Bramanti P., Mazzon E. (2017). The combined strategy of mesenchymal stem cells and tissue-engineered scaffolds for spinal cord injury regeneration. Exp. Ther. Med..

[19] Chudickova M., Vackova I., Urdzikova L.M., Jancova P., Kekulova K., Rehorova M., Turnovcova K., Jendelova P., Kubinova S. (2019). The Effect of Wharton Jelly-Derived Mesenchymal Stromal Cells and Their Conditioned Media in the Treatment of a Rat Spinal Cord Injury. Int. J. Mol. Sci..

[20] Gugliandolo A., Fonticoli L., Trubiani O., Rajan T., Marconi G., Bramanti P., Mazzon E., Pizzicannella J., Diomede F. (2021). Oral Bone Tissue Regeneration: Mesenchymal Stem Cells, Secretome, and Biomaterials. Int. J. Mol. Sci..

[21] El-Sherbiny I.M., Yacoub M.H. (2013). Hydrogel scaffolds for tissue engineering: Progress and challenges. Glob. Cardiol. Sci. Pract..

[22] Silva D., Sousa R., Salgado A. (2021). Hydrogels as delivery systems for spinal cord injury regeneration. Mater. Today Bio.

[23] Radulescu D.-M., Neacsu I.A., Grumezescu A.-M., Andronescu E. (2022). New Insights of Scaffolds Based on Hydrogels in Tissue Engineering. Polymers.

[24] Liu W., Thomopoulos S., Xia Y. (2011). Electrospun Nanofibers for Regenerative Medicine. Adv. Healthc. Mater..

[25] Jiao J., Peng C., Li C., Qi Z., Zhan J., Pan S. (2021). Dual bio-active factors with adhesion function modified electrospun fibrous scaffold for skin wound and infections therapeutics. Sci. Rep..

[26] Kang D.-H., Kim D., Wang S., Song D., Yoon M.-H. (2018). Water-insoluble, nanocrystalline, and hydrogel fibrillar scaffolds for biomedical applications. Polym. J..

[27] Santoro M., Shah S.R., Walker J.L., Mikos A.G. (2016). Poly(lactic acid) nanofibrous scaffolds for tissue engineering. Adv. Drug Deliv. Rev..

[28] Sung Y.K., Kim S.W. (2020). Recent advances in polymeric drug delivery systems. Biomater. Res..

[29] Breen A., O’Brien T., Pandit A. (2009). Fibrin as a Delivery System for Therapeutic Drugs and Biomolecules. Tissue Eng. Part B Rev..

[30] Turnbull G., Clarke J., Picard F., Riches P., Jia L., Han F., Li B., Shu W. (2017). 3D bioactive composite scaffolds for bone tissue engineering. Bioact. Mater..

[31] Chen X., Qi Y.-Y., Wang L.-L., Yin Z., Yin G.-L., Zou X.-H., Ouyang H.-W. (2008). Ligament regeneration using a knitted silk scaffold combined with collagen matrix. Biomaterials.

[32] De Moraes M.A., Nogueira G.M., Weska R.F., Beppu M.M. (2010). Preparation and Characterization of Insoluble Silk Fibroin/Chitosan Blend Films. Polymers.

[33] Zhao S., Wang Z., Chen J., Chen J. (2015). Preparation of heparan sulfate-like polysaccharide and application in stem cell chondrogenic differentiation. Carbohydr. Res..

[34] Chao X., Xu L., Li J., Han Y., Li X., Mao Y., Shang H., Fan Z., Wang H. (2016). Facilitation of facial nerve regeneration using chitosan-*β*-glycerophosphate-nerve growth factor hydrogel. Acta Oto-Laryngol..

[35] Itai S., Suzuki K., Kurashina Y., Kimura H., Amemiya T., Sato K., Nakamura M., Onoe H. (2020). Cell-encapsulated chitosan-collagen hydrogel hybrid nerve guidance conduit for peripheral nerve regeneration. Biomed. Microdevices.

[36] Wang C., Cao X., Zhang Y. (2017). A novel bioactive osteogenesis scaffold delivers ascorbic acid, β-glycerophosphate, and dexamethasone in vivo to promote bone regeneration. Oncotarget.

[37] Kapoor D.N., Bhatia A., Kaur R., Sharma R., Kaur G., Dhawan S. (2015). PLGA: A unique polymer for drug delivery. Ther. Deliv..

[38] Ricard-Blum S. (2011). The Collagen Family. Cold Spring Harb. Perspect. Biol..

[39] Dong C., Lv Y. (2016). Application of Collagen Scaffold in Tissue Engineering: Recent Advances and New Perspectives. Polymers.

[40] Meyer M. (2019). Processing of collagen based biomaterials and the resulting materials properties. Biomed. Eng. Online.

[41] Yannas I.V., Burke J.F., Orgill D.P., Skrabut E.M. (1982). Wound Tissue Can Utilize a Polymeric Template to Synthesize a Functional Extension of Skin. Science.

[42] Karamichos D., Lakshman N., Petroll W.M. (2007). Regulation of Corneal Fibroblast Morphology and Collagen Reorganization by Extracellular Matrix Mechanical Properties. Investig. Ophthalmol. Vis. Sci..

[43] Spill F., Andasari V., Mak M., Kamm R.D., Zaman M.H. (2016). Effects of 3D geometries on cellular gradient sensing and polarization. Phys. Biol..

[44] Wickström S.A., Niessen C.M. (2018). Cell adhesion and mechanics as drivers of tissue organization and differentiation: Local cues for large scale organization. Curr. Opin. Cell Biol..

[45] Xie J., Bao M., Bruekers S.M.C., Huck W.T.S. (2017). Collagen Gels with Different Fibrillar Microarchitectures Elicit Different Cellular Responses. ACS Appl. Mater. Interfaces.

[46] Liu T., Houle J.D., Xu J., Chan B.P., Chew S.Y. (2012). Nanofibrous Collagen Nerve Conduits for Spinal Cord Repair. Tissue Eng. Part A.

[47] Janmey P.A., Winer J.P., Weisel J.W. (2009). Fibrin gels and their clinical and bioengineering applications. J. R. Soc. Interface.

[48] Noori A., Ashrafi S.J., Vaez-Ghaemi R., Hatamian-Zaremi A., Webster T.J. (2017). A review of fibrin and fibrin composites for bone tissue engineering. Int. J. Nanomed..

[49] Aguilar A., Zein N., Harmouch E., Hafdi B., Bornert F., Offner D., Clauss F., Fioretti F., Huck O., Benkirane-Jessel N. (2019). Application of Chitosan in Bone and Dental Engineering. Molecules.

[50] Chatelet C., Damour O., Domard A. (2001). Influence of the degree of acetylation on some biological properties of chitosan films. Biomaterials.

[51] Rodríguez-Vázquez M., Vega-Ruiz B., Ramos-Zúñiga R., Saldaña-Koppel D.A., Quiñones-Olvera L.F. (2015). Chitosan and Its Potential Use as a Scaffold for Tissue Engineering in Regenerative Medicine. BioMed Res. Int..

[52] Oryan A., Sahvieh S. (2017). Effectiveness of chitosan scaffold in skin, bone and cartilage healing. Int. J. Biol. Macromol..

[53] Martins C., Sousa F., Araújo F., Sarmento B. (2017). Functionalizing PLGA and PLGA Derivatives for Drug Delivery and Tissue Regeneration Applications. Adv. Healthc. Mater..

[54] Luderer F., Löbler M., Rohm H.W., Gocke C., Kunna K., Köck K., Kroemer H.K., Weitschies W., Schmitz K.-P., Sternberg K. (2010). Biodegradable Sirolimus-loaded Poly(lactide) Nanoparticles as Drug Delivery System for the Prevention of In-Stent Restenosis in Coronary Stent Application. J. Biomater. Appl..

[55] Caplan A.I. (1991). Mesenchymal stem cells. J. Orthop. Res..

[56] Dominici M., Le Blanc K., Mueller I., Slaper-Cortenbach I., Marini F.C., Krause D.S., Deans R.J., Keating A., Prockop D.J., Horwitz E.M. (2006). Minimal criteria for defining multipotent mesenchymal stromal cells. The International Society for Cellular Therapy position statement. Cytotherapy.

[57] Viswanathan S., Shi Y., Galipeau J., Krampera M., Leblanc K., Martin I., Nolta J., Phinney D.G., Sensebe L. (2019). Mesenchymal stem versus stromal cells: International Society for Cell & Gene Therapy (ISCT^®^) Mesenchymal Stromal Cell committee position statement on nomenclature. Cytotherapy.

[58] Mushahary D., Spittler A., Kasper C., Weber V., Charwat V. (2017). Isolation, cultivation, and characterization of human mesenchymal stem cells. Cytom. Part A.

[59] Lian X.-L., Ji L.-M., Zhang L.-N. (2021). Mannotriose induced differentiation of mesenchymal stem cells into neuron-like cells. J. Integr. Neurosci..

[60] Syková E., Homola A., Mazanec R., Lachmann H., Konrádová L., Kobylka P., Pádr R., Neuwirth J., Komrska V., Vávra V. (2006). Autologous Bone Marrow Transplantation in Patients with Subacute and Chronic Spinal Cord Injury. Cell Transplant..

[61] Lin H., Sohn J., Shen H., Langhans M.T., Tuan R.S. (2019). Bone marrow mesenchymal stem cells: Aging and tissue engineering applications to enhance bone healing. Biomaterials.

[62] Chung C.-S., Fujita N., Kawahara N., Yui S., Nam E., Nishimura R. (2013). A Comparison of Neurosphere Differentiation Potential of Canine Bone Marrow-Derived Mesenchymal Stem Cells and Adipose-Derived Mesenchymal Stem Cells. J. Vet. Med. Sci..

[63] Chen Y.-J., Liu H.-Y., Chang Y.-T., Cheng Y.-H., Mersmann H.J., Kuo W.-H., Ding S.-T. (2016). Isolation and Differentiation of Adipose-Derived Stem Cells from Porcine Subcutaneous Adipose Tissues. J. Vis. Exp..

[64] Prpar Mihevc S., Kokondoska Grgich V., Kopitar A.N., Mohorič L., Majdič G. (2020). Neural differentiation of canine mesenchymal stem cells/multipotent mesenchymal stromal cells. BMC Vet. Res..

[65] Liu J., Yu F., Sun Y., Jiang B., Zhang W., Yang J., Xu G.-T., Liang A., Liu S. (2014). Concise Reviews: Characteristics and Potential Applications of Human Dental Tissue-Derived Mesenchymal Stem Cells. Stem Cells.

[66] Li D., Zou X.-Y., El-Ayachi I., Romero L.O., Yu Z., Iglesias-Linares A., Cordero-Morales J.F., Huang G.T.-J. (2018). Human Dental Pulp Stem Cells and Gingival Mesenchymal Stem Cells Display Action Potential Capacity In Vitro after Neuronogenic Differentiation. Stem Cell Rev. Rep..

[67] Luo L., He Y., Wang X., Key B., Lee B.H., Li H., Ye Q. (2018). Potential Roles of Dental Pulp Stem Cells in Neural Regeneration and Repair. Stem Cells Int..

[68] Vizoso F.J., Eiro N., Cid S., Schneider J., Perez-Fernandez R. (2017). Mesenchymal Stem Cell Secretome: Toward Cell-Free Therapeutic Strategies in Regenerative Medicine. Int. J. Mol. Sci..

[69] Gugliandolo A., Mazzon E. (2021). Dental Mesenchymal Stem Cell Secretome: An Intriguing Approach for Neuroprotection and Neuroregeneration. Int. J. Mol. Sci..

[70] Gao F., Chiu S.M., Motan D.A.L., Zhang Z., Chen L., Ji H.-L., Tse H.-F., Fu Q.-L., Lian Q. (2016). Mesenchymal stem cells and immunomodulation: Current status and future prospects. Cell Death Dis..

[71] Krabbe C., Zimmer J., Meyer M. (2005). Neural transdifferentiation of mesenchymal stem cells—A critical review. APMIS.

[72] Reilly G.C., Engler A.J. (2010). Intrinsic extracellular matrix properties regulate stem cell differentiation. J. Biomech..

[73] Naqvi S.M., McNamara L.M. (2020). Stem Cell Mechanobiology and the Role of Biomaterials in Governing Mechanotransduction and Matrix Production for Tissue Regeneration. Front. Bioeng. Biotechnol..

[74] Li M., Mei X., Lv S., Zhang Z., Xu J., Sun D., Xu J., He X., Chi G., Li Y. (2018). Rat vibrissa dermal papilla cells promote healing of spinal cord injury following transplantation. Exp. Ther. Med..

[75] Peng Z., Gao W., Yue B., Jiang J., Gu Y., Dai J., Chen L., Shi Q. (2016). Promotion of neurological recovery in rat spinal cord injury by mesenchymal stem cells loaded on nerve-guided collagen scaffold through increasing alternatively activated macrophage polarization. J. Tissue Eng. Regen. Med..

[76] Liu D., Li X., Xiao Z., Yin W., Zhao Y., Tan J., Chen B., Jiang X., Dai J. (2019). Different functional bio-scaffolds share similar neurological mechanism to promote locomotor recovery of canines with complete spinal cord injury. Biomaterials.

[77] Deng W.-S., Ma K., Liang B., Liu X.-Y., Xu H.-Y., Zhang J., Shi H.-Y., Sun H.-T., Chen X.Y., Zhang S. (2020). Collagen scaffold combined with human umbilical cord-mesenchymal stem cells transplantation for acute complete spinal cord injury. Neural Regen. Res..

[78] Deng W.-S., Liu X.-Y., Ma K., Liang B., Liu Y.-F., Wang R.-J., Chen X.-Y., Zhang S. (2021). Recovery of motor function in rats with complete spinal cord injury following implantation of collagen/silk fibroin scaffold combined with human umbilical cord-mesenchymal stem cells. Rev. Assoc. Med. Bras..

[79] Deng W.-S., Yang K., Liang B., Liu Y.-F., Chen X.-Y., Zhang S. (2021). Collagen/heparin sulfate scaffold combined with mesenchymal stem cells treatment for canines with spinal cord injury: A pilot feasibility study. J. Orthop. Surg..

[80] Zou Y., Zhao Y., Xiao Z., Chen B., Ma D., Shen H., Gu R., Dai J. (2020). Comparison of Regenerative Effects of Transplanting Three-Dimensional Longitudinal Scaffold Loaded-Human Mesenchymal Stem Cells and Human Neural Stem Cells on Spinal Cord Completely Transected Rats. ACS Biomater. Sci. Eng..

[81] Wang N., Xiao Z., Zhao Y., Wang B., Li X., Li J., Dai J. (2018). Collagen scaffold combined with human umbilical cord-derived mesenchymal stem cells promote functional recovery after scar resection in rats with chronic spinal cord injury. J. Tissue Eng. Regen. Med..

[82] Han S., Xiao Z., Li X., Zhao H., Wang B., Qiu Z., Mei X., Xu B., Fan C., Chen B. (2018). Human placenta-derived mesenchymal stem cells loaded on linear ordered collagen scaffold improves functional recovery after completely transected spinal cord injury in canine. Sci. China Life Sci..

[83] Sabino L., Maria C., Luca L., Valerio V., Edda F., Giacomo R., Gloria I., Juan G., Antonio C. (2018). Engraftment, neuroglial transdifferentiation and behavioral recovery after complete spinal cord transection in rats. Surg. Neurol. Int..

[84] Chandrababu K., Sreelatha H.V., Sudhadevi T., Anil A., Arumugam S., Krishnan L.K. (2021). In vivo neural tissue engineering using adipose-derived mesenchymal stem cells and fibrin matrix. J. Spinal Cord Med..

[85] Mukhamedshina Y.O., Akhmetzyanova E., Kostennikov A., Zakirova E.Y., Galieva L.R., Garanina E.E., Rogozin A.A., Kiassov A.P., Rizvanov A. (2018). Adipose-Derived Mesenchymal Stem Cell Application Combined With Fibrin Matrix Promotes Structural and Functional Recovery Following Spinal Cord Injury in Rats. Front. Pharmacol..

[86] García E., Rodríguez-Barrera R., Buzoianu-Anguiano V., Flores-Romero A., Malagón-Axotla E., Guerrero-Godinez M., De la Cruz-Castillo E., Castillo-Carvajal L., Rivas-Gonzalez M., Santiago-Tovar P. (2019). Use of a combination strategy to improve neuroprotection and neuroregeneration in a rat model of acute spinal cord injury. Neural Regen. Res..

[87] Ibarra A., Mendieta-Arbesú E., Suarez-Meade P., Vences E.E.G., Martiñón S., Rodriguez-Barrera R., Lomelí J., Flores-Romero A., Silva-García R., Buzoianu-Anguiano V. (2019). Motor Recovery after Chronic Spinal Cord Transection in Rats: A Proof-of-Concept Study Evaluating a Combined Strategy. CNS Neurol. Disord. Drug Targets.

[88] Rodríguez-Barrera R., Flores-Romero A., Buzoianu-Anguiano V., Garcia E., Soria-Zavala K., Incontri-Abraham D., Garibay-López M., Whaley J.J.J.-V., Ibarra A. (2020). Use of a Combination Strategy to Improve Morphological and Functional Recovery in Rats With Chronic Spinal Cord Injury. Front. Neurol..

[89] Mukhamedshina Y., Shulman I., Ogurcov S., Kostennikov A., Zakirova E., Akhmetzyanova E., Rogozhin A., Masgutova G., James V., Masgutov R. (2019). Mesenchymal Stem Cell Therapy for Spinal Cord Contusion: A Comparative Study on Small and Large Animal Models. Biomolecules.

[90] Yao S., He F., Cao Z., Sun Z., Chen Y., Zhao H., Yu X., Wang X., Yang Y., Rosei F. (2020). Mesenchymal Stem Cell-Laden Hydrogel Microfibers for Promoting Nerve Fiber Regeneration in Long-Distance Spinal Cord Transection Injury. ACS Biomater. Sci. Eng..

[91] Spejo A.B., Chiarotto G.B., Ferreira A.D.F., Gomes D.A., Ferreira R.S., Barraviera B., Oliveira A.L.R. (2018). Neuroprotection and immunomodulation following intraspinal axotomy of motoneurons by treatment with adult mesenchymal stem cells. J. Neuroinflamm..

[92] Basak A.T., Cakici N., Bozkurt G., Purali N., Denkbas E.B., Korkusuz P., Cetinkaya D.U. (2021). Chitosan channels with stuffed mesenchyme originated stem/progenitor cells for renovate axonal regeneration in complete spinal cord transection. Turk. Neurosurg..

[93] Zhang J., Cheng T., Chen Y., Gao F., Guan F., Yao M.-H. (2020). A chitosan-based thermosensitive scaffold loaded with bone marrow-derived mesenchymal stem cells promotes motor function recovery in spinal cord injured mice. Biomed. Mater..

[94] Ji W.-C., Li M., Jiang W.-T., Ma X., Li J. (2020). Protective effect of brain-derived neurotrophic factor and neurotrophin-3 overexpression by adipose-derived stem cells combined with silk fibroin/chitosan scaffold in spinal cord injury. Neurol. Res..

[95] Alizadeh A., Moradi L., Katebi M., Ai J., Azami M., Moradveisi B., Ostad S.N. (2020). Delivery of injectable thermo-sensitive hydrogel releasing nerve growth factor for spinal cord regeneration in rat animal model. J. Tissue Viability.

[96] Huang C., Liu Y., Ding J., Dai Y., Le L., Wang L., Ding E., Yang J. (2021). Thermosensitive quaternized chitosan hydrogel scaffolds promote neural differentiation in bone marrow mesenchymal stem cells and functional recovery in a rat spinal cord injury model. Cell Tissue Res..

[97] Boido M., Ghibaudi M., Gentile P., Favaro E., Fusaro R., Tonda-Turo C. (2019). Chitosan-based hydrogel to support the paracrine activity of mesenchymal stem cells in spinal cord injury treatment. Sci. Rep..

[98] Song X., Xu Y., Wu J., Shao H., Gao J., Feng X., Gu J. (2020). A sandwich structured drug delivery composite membrane for improved recovery after spinal cord injury under longtime controlled release. Colloids Surf. B Biointerfaces.

[99] Xu Z.-X., Zhang L.-Q., Zhou Y.-N., Chen X.-M., Xu W.-H. (2020). Histological and functional outcomes in a rat model of hemisected spinal cord with sustained VEGF/NT-3 release from tissue-engineered grafts. Artif. Cells Nanomed. Biotechnol..

[100] Ropper A.E., Thakor D.K., Han I., Yu D., Zeng X., Anderson J.E., Aljuboori Z., Kim S.-W., Wang H., Sidman R.L. (2017). Defining recovery neurobiology of injured spinal cord by synthetic matrix-assisted hMSC implantation. Proc. Natl. Acad. Sci. USA.

[101] Yang E.-Z., Zhang G.-W., Xu J.-G., Chen S., Wang H., Cao L.-L., Liang B., Lian X.-F. (2017). Multichannel polymer scaffold seeded with activated Schwann cells and bone mesenchymal stem cells improves axonal regeneration and functional recovery after rat spinal cord injury. Acta Pharmacol. Sin..

[102] Han I.-B., Thakor D.K., Ropper A.E., Yu D., Wang L., Kabatas S., Zeng X., Kim S.-W., Zafonte R.D., Teng Y.D. (2019). Physical impacts of PLGA scaffolding on hMSCs: Recovery neurobiology insight for implant design to treat spinal cord injury. Exp. Neurol..

[103] Zhao Y., Tang F., Xiao Z., Han G., Wang N., Yin N., Chen B., Jiang X., Yun C., Han W. (2017). Clinical Study of NeuroRegen Scaffold Combined with Human Mesenchymal Stem Cells for the Repair of Chronic Complete Spinal Cord Injury. Cell Transplant..

[104] Xiao Z., Tang F., Zhao Y., Han G., Yin N., Li X., Chen B., Han S., Jiang X., Yun C. (2018). Significant Improvement of Acute Complete Spinal Cord Injury Patients Diagnosed by a Combined Criteria Implanted with NeuroRegen Scaffolds and Mesenchymal Stem Cells. Cell Transplant..

[105] Tang F., Tang J., Zhao Y., Zhang J., Xiao Z., Chen B., Han G., Yin N., Jiang X., Zhao C. (2021). Long-term clinical observation of patients with acute and chronic complete spinal cord injury after transplantation of NeuroRegen scaffold. Sci. China Life Sci..

